# Adjunctive use of essential oils following scaling and root planing –a randomized clinical trial

**DOI:** 10.1186/s12906-016-1117-x

**Published:** 2016-06-07

**Authors:** Mohammad Fallah Azad, Andreas Schwiertz, Holger F. R. Jentsch

**Affiliations:** Private dental practice, Hamburg, Germany; Institute of Microecology, Herborn, Germany; Centre for Periodontology, Department for Cariology, Endodontology and Periodontology, University Hospital of Leipzig, Liebigstr. 12, Haus 1, D-04103 Leipzig, Germany

**Keywords:** Chronic periodontitis, Essential oils, Periodontopathogens, Scaling and root planing

## Abstract

**Background:**

Hitherto no study has been published on the effect of the adjunctive administration of essential oils following scaling and root planing (SRP). This study describes the effect of a mouthrinse consisting of essential oils (*Cymbopogon flexuosus*, *Thymus zygis* and *Rosmarinus officinalis*) following SRP by clinical and microbiological variables in patients with generalized moderate chronic periodontitis.

**Methods:**

Forty-six patients (aged 40–65 years) with moderate chronic periodontitis were randomized in a double-blind study and rinsed their oral cavity following SRP with an essential oil mouthrinse (*n*  =  23) or placebo (*n*  =  23) for 14 days. Probing depth (PD), attachment level (AL), bleeding on probing (BOP) and modified sulcus bleeding index (SBI) were recorded at baseline and after 3 and 6 months. Subgingival plaque was taken for assessment of major bacteria associated with periodontitis.

**Results:**

AL, PD, BOP and SBI were significantly improved in both groups after three (*p*   <   0.001) and 6 months (*p*   ≤   0.015). AL improved significantly better in the test than in the control group after 3 and 6 months (*p* < 0.001), so did PD after three months in the tendency (*p*  =  0.1). BOP improved better in the test group after 3 months (*p*  =  0.065). Numbers of *Treponema denticola* (*p*  =  0.044) and *Fusobacterium nucleatum* (*p*  =  0.029) decreased more in the test than in the control group after 3 months, those of *Tannerella forsythia* after 6 months (*p*  =  0.039). *Prevotella micra* (p  <  0.001, *p*  =  0.035) and *Campylobacter rectus* (*p*  =  0.002 , *p*  =  0.012) decreased significantly in both groups after 3 months.

**Conclusions:**

The adjunctive use of a mouthrinse containing essential oils following SRP has a positive effect on clinical variables and on bacterial levels in the subgingival biofilm.

**Trial registration:**

332-12-24092012, DRKS 00009387, German Clinical Trials Register, Freiburg i. Br., 16.09.2015

**Electronic supplementary material:**

The online version of this article (doi:10.1186/s12906-016-1117-x) contains supplementary material, which is available to authorized users.

## Background

Periodontitis is a multifactorial disease, meaning that a single treatment approach is often not enough to combat the bacterial colonisation [1]. The multi-causal etiology of periodontal disease requires interdisciplinary treatment concepts and the selection of a therapy that focuses on the microbiological nature of the disease [2].

Currently, mechanical cleaning via scaling and root planing (SRP) represents the method of choice [3], which, through the removal of subgingival biofilm from the root surface, leads to greater reduction of periodontopathogenic microorganisms. However, studies have shown that remaining bacteria cannot be fully eliminated and the adjunctive administration of antimicrobial substances is necessary [4, 5].

The variety of possible adjuvant periodontitis treatment procedures includes local and systemic antibiotics [6], photodynamic therapy [7, 8], Er:YAG lasers [9], povidone-iodine [10, 11] as well as chlorhexidine digluconate [12–14].

To improve the outcome of SRP antibiotics are used in severe forms of periodontitis [15, 16] and mostly chlorhexidine rinsing is executed in the first days after SRP [17, 18].

In the last two decades essential oils were extensively tested regarding their antibacterial properties against a broad spectrum of bacteria. Hammer et al. [19] investigated also in vitro how tea tree oils alter the permeability and membrane fluidity of different yeasts including *Candida albicans*. Based on their results, it was assumed that essential oils may have antimicrobial activity by influencing bacterial targets involved in cytoplasmatic and membrane metabolism. Other in vitro studies displayed that monoterpenes are important components of essential oils and increase fluidity and permeability of membranes and thus disturb membrane proteins, resulting in the inhibition of cell respiration and confusion of ion transport processes [20].

*Cymbopogon flexuosus* oil (lemongrass oil) has in vitro a strong antimicrobial and antifungal activity and inhibits very efficiently early biofilm formation [21, 22]. This oil elicites morphological changes like filamentation, inhibition of septum formation, spheroplast formation, as well as lysis and development of abnormally shaped cells. In vitro the incorporation of diaminopimelic acid into the cell wall murein of strain W7, was inhibited in a dose dependent way [23]. Thymus zygis oil has also a very strong antibacterial activity tested against different bacterial species and modulates inflammatory cytokines like IL-1β, TNFα, IL-6 and IL-10 in vitro [24]. Antioxidative effects of Rosmarinus officinalis leaf oil have also been described [25].

The aim of this prospective randomized study was to identify, if the adjunctive use of a mouthwash containing three essential oils (Cymbopogon flexuosus oil, Thymus Zygis oil, Rosmarinus officinalis) in the first days following SRP can improve the clinical results of SRP and has effects on the composition of the subgingival biofilm. Our hypothesis was to test, whether there is a significant difference between the adjunctive treatment with or without essential oil mouthwash, regarding changes of the clinical parameters PD, AL and BOP after 6 months.

## Methods

### Study design

Following approval of the study by the Ethics Commission (332-12-24092012, see Additional file 1) of the Medical Faculty at the University of Leipzig, the voluntary participants were recruited from the patient’s pool of a private dental practice (M. F.). After extensive consultation and signing the informed consent the randomized double-blinded study started in April 2013 and ended in September 2014. The study was conducted in full accordance with the principles outlined in the Declaration of Helsinki, as revised in 2000. All treatment was performed by the same dentist. To avoid bias, another investigator blinded the treatment collected plaque and assessed the clinical data.

### Patients

Patients were included in the study by fulfilling the following criteria: being between 40 and 65 years old, having a generalised moderate chronic periodontitis [26] having a minimum of 20 teeth, probing depth (PD) between 4 and 6 mm, no periodontal treatment within the past year, and no antibiotic therapy within the last 6 months. The interproximal plaque index (API, [27]) was required to be ≤ 35 % after two initial prophylaxis and instruction sessions. Results of the API ≤ 35 % reflect a sufficient hygiene to start with periodontal therapy [27]. Pregnancy, breastfeeding and allergy to the ingredients of the herbal distillate products were exclusion criteria.

The 50 patients were allocated into a test group consisting of 25 and a control group of 25 participants with the use of a computer-generated randomization table. All patients were asked for smoking (non-smokers, former smokers, smokers.).

The clinical variables PD, attachment level (AL) and bleeding on probing (BOP) of all teeth were determined in a six-point measurement per tooth (mesiobuccal, buccal, distobuccal, mesiooral, oral and distooral) with a manual periodontal probe (PCP-UNC 15, Hu-Friedy Manufacturing Co., Chicago, IL, USA) at three appointments: before SRP (baseline), after three and 6 months. The modified sulcus bleeding index (SBI) was recorded. At the same time, samples of the subgingival biofilm were taken at one site per quadrant with PD 4 - 6 mm.

For sampling of the subgingival biofilm, endodontic paper points (ISO 60, Roeko GmbH, Langenau, Germany) were inserted into the pocket until resistance was felt and were left in place for 15 s. The strips and points were stored as a pooled sample at - 20° C after sampling and immediately transferred to the laboratory (Institut Mikroökologie, Herborn, Germany) for analysis.

### Therapy and follow-Up treatment

Under local anaesthesia with articaine hydrochloride/epinephrine hydrochloride (Ultracain D-S, Sanofi-Aventis, Frankfurt/Main, Germany), the participants received full-mouth scaling and root planing (SRP) in two sessions carried out within 24 h using hand and ultrasonic instruments. All patients used a chlorhexidine digluconate mouthwash (Chlorhexamed forte 0.2 %, GlaxoSmithKline Healthcare, Bühl, Germany) for one minute twice daily during the first seven days after SRP and performed carefully normal oral hygiene with toothbrush and interdental brushes.

At the end of the SRP, the volunteers in the test group were requested to rinse their oral cavity with Parodolium® mouthwash (Symbio Vaccin, Herborn, Germany). The composition of the mouthrinse was as follows: *Cymbopogon flexuosus* oil, *Thymus zygis* oil, *Rosmarinus officinalis* leaf oil, PEG-40-hydrogenated-castor-oils-emulsifier, aqua (0.5 %, 0.5 %, 0.5 %, 6 %, 92.5 %). During the first two weeks after SRP the oral cavity was rinsed with five drops in a glass of water for sixty second twice daily. In the control group a blinded placebo provided by the manufacturer was used for rinsing. The placebo was composed of the emulsifier and water. The patients were controlled for exact drug use by a phone call after three and 10 days. Both mouthwashes were labelled and separately used by the instructed patient. Appointments of supportive periodontal therapy took place 3 months after SRP. Professional tooth cleaning and reinforcement of oral hygiene were performed.

### Microbiological analysis

For microbiological analysis, DNA was extracted and cleaned using the NucliSENS® easyMag® (Biomerieux, Nürtingen, Germany) according to the manufacturer’s description. The analysis was performed using the ParoCheck® kit (Greiner Bio-One, Frickenhausen, Germany, Henne et al. 2014 [28]. The test is able to identify up to 20 periodontopathogenic bacterial species after two PCR runs and a subsequent reverse hybridization. For the study the following bacteria were analysed semiquantitatively: *Tannerella forsythia, Porphyromonas gingivalis, Treponema denticola, Prevotella intermedia, Parvimonas micra, Campylobacter rectus/showae, Fusubacterium nucleatum, Aggregatibacter actinomycetemcomitans, Eikenella corrodens* and *Actinomyces viscosus.*

The results were categorized as follows: category 0 (signal-noise-ratio/SNR: 0–12, negative), category 1 (SNR: 13–21, norm range), category 2 (SNR: 21–40), category 3 (SNR: 40–200) and category 4 (SNR: >200). Category 0 and 1 were merged together, being denoted as category 0–1.

### Statistical analysis

The statistical analysis was carried out with assistance of SPSS, Version 22.0 (SPSS Inc., NY, U.S.A.). The null hypothesis was that there are no statistically significant differences between the test and control group, regarding clinical parameters PD, AL and BOP. Change of PD at the 6-months appointment was set as the primary outcome and used to determine sample size. A mean difference of 1 mm in observed PD with a standard deviation of 1 mm between two groups or two examination dates would require ≥ 16 patients per group in order to detect a significant difference (*p* ≤ 0.05) with a test power of 80 % (GraphPad StatMate v.2.0 for Mac, GraphPad Software, San Diego, CA, U.S.A.). We started with 25 Patients to compensate for possible dropouts. Secondary outcome variables were changes of PD after 3 months, in the number of sites with PD ≥ 5 mm, occurrence of BOP, mean AL as well as the counts of selected pathogenic bacteria associated with periodontitis after three and 6 months. The statistical unit was the individual patient. For both intra- and inter-group comparisons, non-parametric tests (Wilcoxon test for paired samples and Mann Whitney U - test, respectively) and *χ*^2^ - test were used. A level of α ≤ 0.05 was considered as being significant.

## Results

**Fig. 1:**
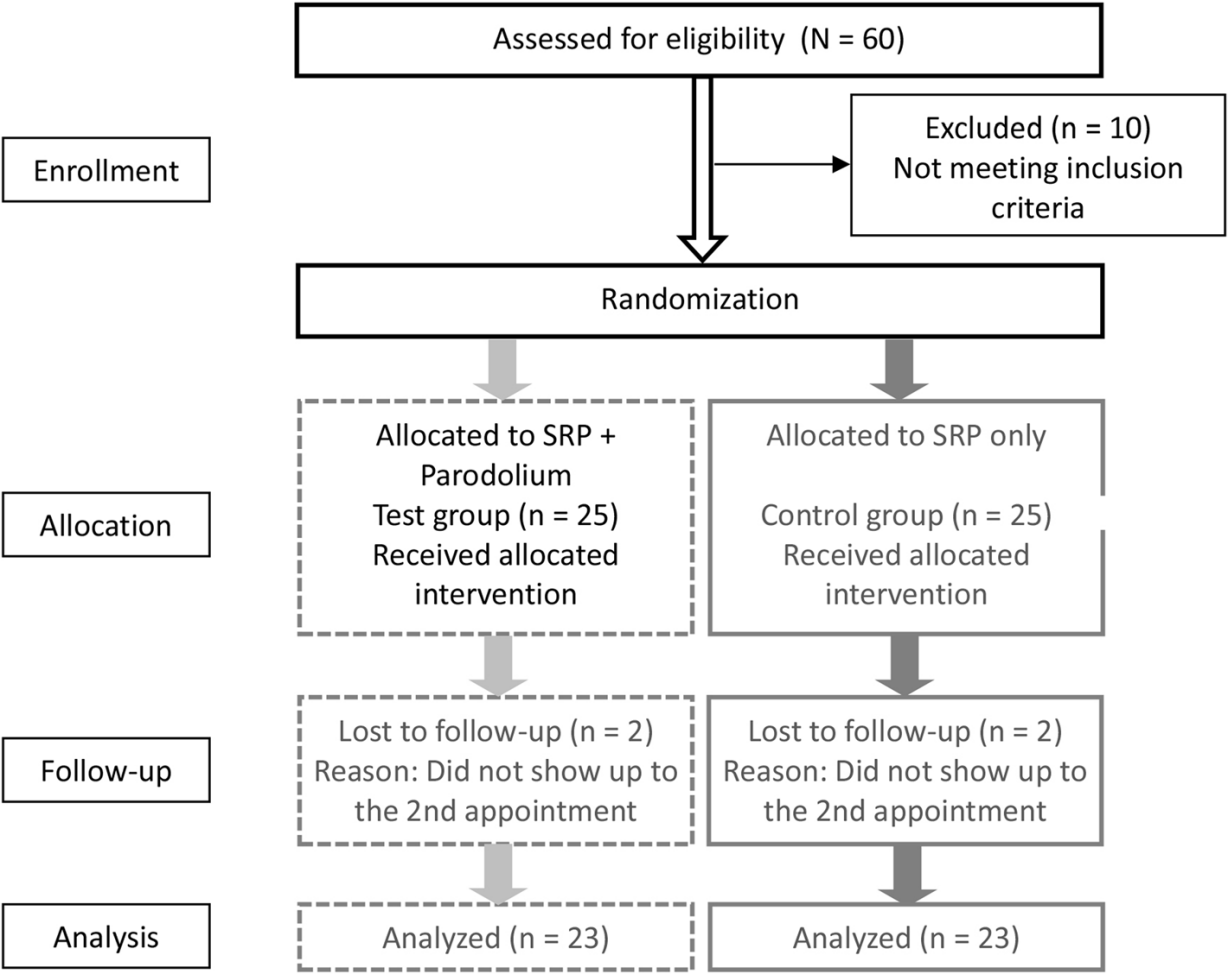
Flowchart (adapted to [29]) of the study analyzing the effect of herbal distillates as an adjunct after SRP

Figure 1 presents the study flow adapted to Moher et al. [29]. 60 patients were assessed for eligibility, 10 patients were excluded. There were two drop outs in each group, these four patients did not attend the second appointment. Finally, data from 23 patients (11 male, 12 female; mean age 50.6 years, range 43 – 62 years) in the test group and data from 23 patients in the control group (13 male, 10 female, mean age 50.9 years, range 41 – 60 years) were evaluated. The demographic data of these 46 patients are summarized in Table 1. No adverse effects of the essential oil based mouthwash were observed during the study. The compliance of the patients was improved during initial therapy before SRP, API values of 18 % and 21 % were achieved in the test and control groups, respectively.

**Table 1:** Clinical results at baseline

Variables		test	control	P (*U* test)
n		23	23	
Age	(years)	50.6 ± 5.8	50.9 ± 6.9	
Males	(n)	11	13	
Females	(n)	12	10	
Smoker	(n)	8	8	
PD	(mm)	3.3 ± 0.4	3.3 ± 0.3	0.965
AL	(mm)	3.5 ± 0.2	3.4 ± 0.3	0.947
BOP	(%)	16.5 ± 4	16.7 ± 5.5	0.724
API	(%)	18.3 ± 8.2	21.7 ± 5.6	0.094
SBI	(%)	18.7 ± 6.5	18.1 ± 5.6	0.869

The changes of the clinical variables are presented in Table 2. Significant improvements of AL, PD, BOP and SBI occurred in both groups after three (*p*  <  0.001) and 6 months (*p*  ≤  0.015). AL was significantly better in the test group after 3 and 6 months (2.5 and 2.6 mm vs. 2.8 mm, *p*  <  0.001), so did PD after 3 months in the tendency (*p*  =  0.1). BOP was significantly lower in the test group after three months (6.0 vs. 9.0, *p* = 0.027). The improvement of BOP was also better in the test group after three months (*p*  =  0.065). There was no significant difference between the groups at SBI.

**Table 2:** Clinical variables (median, 25 and 75 percentile, mean ± SD at baseline, after 3 and 6 months, differences and statistical analysis

N test group = 23 N control group = 23	Baseline	3 months	6 months	Difference t0-t3	Difference t0-t6
BoP (%)					
Test Control	17.0 (13.0, 19.0) *** 16.0 (13.0, 19.0) ***	6.0 (4.0, 9.0) *** †† 9.0 (5.0, 12.0) ***	12.0 (9.0, 14.0) *** 11.0 (8.0, 17.0) ***	10.0 (8.0, 12.0) *** † 9.0 (6.0, 11.0) ***	5.0 (3.0, 8.0) *** 4.0 (2.0, 7.0) ***
Test Control	16.5 ± 4.0 16.7 ± 5.5	6.2 ± 3.0 8.7 ± 3.7	11.5 ± 4.9 13.0 ± 6.7	10.3 ± 3.1 8.0 ± 4.0	5.0 ± 4.0 3.7 ± 5.6
SBI (%)					
Test group Control group	18.0 (14.0, 23.0) *** 16.0 (14.0, 21.0) ***	12.0 (8.0, 15.0) *** 11.0 (7.0, 15.0) ***	13.0 (11.0, 18.0) *** 13.0 (11.0, 15.0) ***	7.0 (4.0, 11.0) *** 8.0 (4.0, 12.0) ***	4.0 (0.0, 8.0) ** 4.0 (3.0, 8.0) **
Test Control	18.7 ± 6.5 18.1 ± 5.6	11.8 ± 4.4 10.3 ± 4.3	13.4 ± 5.2 13.4 ± 3.8	6.8 ± 5.2 7.8 ± 5.4	5.2 ± 5.7 4.7 ± 5.9
Mean PD (mm)					
Test Control	3.3 (3.1, 3.6) *** 3.2 (3.1, 3.5) ***	2.1 (2.0, 2.2) *** †† 2.2 (2.0, 2.5) ***	2.2 (2.0, 2.3) *** †† 2.3 (2.1, 2.5) ***	1.2 (0.8, 1.2) *** † 1.0 (0.9, 1.5) ***	1.1 (0.8, 1.2) *** 1.0 (0.8, 1.5) ***
Test Control	3.3 ± 0.4 3.3 ± 0.3	2.1 ± 0.3 2.3 ± 0.2	2.2 ± 0.2 2.3 ± 0.3	1.2 ± 0.3 1.0 ± 0.3	1.1 ± 0.3 1.0 ± 0.3
Mean AL (mm)					
Test group Control group	3.5 (3.3, 3.7) *** 3.5 (3.3, 3.7) ***	2.5 (2.4, 2.6) *** †† 2.8 (2.5, 2.9) ***	2.6 (2.5, 2.7) *** †† 2.8 (2.7, 3.0) ***	0.9 (0.8, 1.1) *** ††† 0.7 (0.6, 0.8) ***	0.9 (0.7, 1.0) *** ††† 0.6 (0.5, 0.7) ***
Test Control	3.5 ± 0.2 3.4 ± 0.3	2.5 ± 0.3 2.7 ± 0.3	2.6 ± 0.2 2.8 ± 0.3	1.0 ± 0.3 0.7 ± 0.2	0.9 ± 0.2 0.6 ± 0.2
Sites PD > 5 mm (n)					
Test Control	18.0 (13.0,27.0) 19.0 (9.0, 25.0)	0.0 (0.0, 2.0) 1.0 (0.0, 5.0)	0.0 (0.0, 2.0) 2.0 (0.0, 6.0)	17.0 (13.0, 26.0) 17.0 (8.0, 22.0)	18.0 (13.0, 25.0) 16.0 (7.0, 22.0)
Test Control	18.7 ± 10.3 19.4 ± 13.0	1.1 ± 1.7 2.7 ± 3.3	1.3 ± 2.2 3.4 ± 4.2	17.6 ± 9.2 16.7 ± 10.2	17.4 ± 8.7 16.0 ± 9.9
Mean sites PD > 5 mm (n)					
Test Control	5.3 (5.0, 5.5) 5.3 (5.0, 5.5)	3.0 (2.8, 3.3) 3,0 (2.8, 3.3)	3.0 (2.8, 3.5) 3.0 (2.8, 3.3)	2.5 (2.3, 2.5) 2.3 (2.0, 2.5)	2.3 (2.0, 2.5) 2.3 (2.0, 2.3)
Test Control	5.3 ± 0.4 5.3 ± 0.4	3.0 ± 0.4 3.0 ± 0.3	3.1 ± 0.4 3.1 ± 0.3	2.3 ± 0.2 2.3 ± 0.3	2.2 ± 0.3 2.2 ± 0.4
Sites AL > 5 mm (n)					
Test Control	21.0 (14.0, 31.0) 21.0 (12.0, 27.0)	2.0 (0.0, 3.0) 2.0 (0.0, 6.0)	1.0 (0.0, 3.0) 3.0 (0.0, 8.0)	21.0 (14.0, 27.0) 16.0 (12.0, 24.0)	21.0 (14.0, 27.0) 16.0 (9.0, 23.0)
Test Control	21.3 ± 11.5 22.3 ± 14.7	2.1 ± 3.3 3.4 ± 4.2	2.4 ± 3.7 4.3 ± 4.4	19.2 ± 9.5 18.9 ± 11.7	18.9 ± 9.1 18.0 ± 11.7
Mean sites AL > 5 mm (n)					
Test Control	4.0 (4.0, 4.3) 4.0 (4.0, 4.3)	2.8 (2.8, 3.0) 3.0 (2.8, 3.0)	3.0 (3.0, 3.0) 3.0 (3.0, 3.3)	1.3 (1.3, 1.5) 1.3 (1.0, 1.5)	1.0 (1.0, 1.3) 1.0 (0.8, 1.3)
Test Control	4.2 ± 0.3 4.1 ± 0.2	2.9 ± 0.2 2.9 ± 0.3	3.1 ± 0.2 3.1 ± 0.2	1.3 ± 0.2 1.2 ± 0.3	1.1 ± 0.3 1.0 ± 0.2

Wilcoxon signed rank test: significant longitudinal changes t3 - t0, t6 - t0 within each group

** 0.01  <  *p*  <  0.05, *** *p*  <  0.001

Mann Whitney test between the groups (test-control): † 0.05  <  *p*  <  0.1, †† 0.01  <  *p*  <  0.05, †††    <  0.001

The results for the microbiological analysis are given in Table 3, which comprises all the 10 investigated bacteria.

**Table 3:** Prevalence (%) of bacteria at baseline, after 3 and 6 months and significance of differences

Species	Cat.	Baseline		3 months		6 months		Sig. Δ_t0-t3_ inter	Sig. Δ_t0-t6_ inter	Sig. Δ_t0-t3_ intra	Sig. Δ_t0-t6_ intra
		test	control	test	control	test	control				
		*N* = 23	*N* = 23	*N* = 23	*N* = 23	*N* = 23	*N* = 23				
*A. act.*	0-1	82.6	91.3	87	91.3	82.6	91.3	*p* = 0.156	*p* = 0.221	p_t_ = 0.625	p_t_ = 0.375
	2	4.3	0	0	4.3	0	4.3				
	3 4	13.0 0	0 8.7	4.3 8.7	0 4.3	8.7 8.7	0 4.3			p_c_ = xxx	p_c_ = xxx
*P. ging.*	0-1	60.8	43.5	73.9	60.9	52.1	39.1	*p* = 0.693	*p* = 0.849	p_t_ = 0.289	p_t_ = 0.687
	2	4.3	0	4.3	13.0	0	0				
	3 4	0 34.8	8.7 47.8	8.7 13.0	4.3 21.7	17.4 30.4	17.4 43.5			p_c_ = 0.109	p_c_ = 1.000
*T. dent.*	0-1	13	0	30.4	13	4.3	0	*p* = 0.044	*p* = 0.224	p_t_ = 0.057	p_t_ = 0.549
	2	4.3	4.3	8.7	17.4	8.7	0				
	3 4	0 82.6	17.4 78.3	21.7 39.1	39.1 30.4	26.1 60.9	21.7 78.3			p_c_ = 0.004	p_c_ = 1.000
*T. fors.*	0-1	8.7	4.3	26	13	13	4.3	*p* = 0.632	*p* = 0.039	p_t_ = 0.012	p_t_ = 0.754
	2	4.3	0	13	4.3	0	0				
	3 4	8.7 78.3	0 95.7	13 47.8	21.7 60.9	26.1 60.9	4.3 91.3			p_c_ = 0.008	p_c_ = xxx
*F. nucl.*	0-1	4.3	0	0	4.3	4.3	0	*p* = 0.029	*p* = 0.179	p_t_ = 0.344	p_t_ = 1.000
	2	8.7	0	13.0	4.3	4.3	0				
	3 4	8.7 78.3	4.3 95.7	26.1 60.9	0 91.3	17.4 73.9	8.7 91.3			p_c_ = 1.000	p_c_ = xxx
*P. micra*	0-1	34.7	4.3	52.2	39.1	26.1	13	*p* = 0.127	*p* = 0.155	p_t_ = 0.035	p_t_ = 1.000
	2	4.3	13.0	8.7	8.7	21.7	13				
	3 4	30.4 30.4	47.8 34.8	34.8 4.3	52.2 0	39.1 13.0	65.2 8.7			p_c_ < 0.001	p_c_ = 0.210
*C. rectus*	0-1	21.7	30.4	56.5	56.5	39.1	39.1	0.507	0.142	p_t_ = 0.002	p_t_ = 0.115
	2	8.7	4.3	21.7	4.3	30.4	13.0				
	3 4	56.5 13.0	47.8 17.4	21.7 0	39.1 0	26.1 4.3	43.5 4.3			p_c_ = 0.012	p_c_ = 0.227
*P. interm.*	0-1	65.2	39.1	78.2	56.5	65.2	34.7	*p* = 0.537	*p* = 0.605	p_t_ = 0.508	p_t_ = 1.000
	2	4.3	0	4.3	21.7	4.3	0				
	3 4	8.7 21.7	8.7 52.2	4.3 13.0	4.3 17.4	21.7 8.7	39.1 26.1			p_c_ = 0.039	p_c_ = 0.344
*E. corrod.*	0-1	52.2	56.5	73.9	78.2	34.7	65.2	*p* = 0.189	*p* = 0.364	p_t_ = 1.000	p_t_ = 0.302
	2	13.0	13.0	4.3	8.7	21.7	4.3				
	3 4	21.7 13.0	21.7 8.7	13.0 8.7	13.0 0.0	34.8 8.7	21.7 8.7			p_c_ = 0.146	p_c_ = 0.549
*A. viscosus*	0-1	21.7	4.3	17.3	21.7	8.7	4.3	*p* = 0.411	*p* = 0.067	p_t_ = 0.118	p_t_ = 0.424
	2	17.4	8.7	8.7	8.7	13.0	30.4				
	3 4	47.8 13.0	56.5 30.4	39.1 34.8	52.2 17.4	56.5 21.7	43.5 21.7			p_c_ = 0.210	p_c_ = 0.267

Categories 0 and 1 are summarized and denoted as “0-1”

The depicted significance-values are devided in inter-group (test vs. control) and intra-group (the same group at two different times) comparison

The intragroup significances are devided into those of the test group (p_t_) and control group (p_c_)

*C. rectus* was significantly reduced in both groups after three months (p_t_   =  0.002, p_c_  =  0.012), so did *P. micra* (p_t_   =  0.035, p_c_  <  0.001) and T. forsythia (p_t_   =  0.012, p_c_  =  0.008).

*T. denticola* was also reduced in both groups after 3 months (p_t_   =  0.057, p_c_  =  0.004) but with significantly higher reduction in the test group, (*p*  =  0.044). *F. nucleatum* was significantly more reduced in the test group than in the control group after three months (*p*  =  0.029). After six months *T. forsythia* was still reduced in the test group, but not in the control group, with significant intergroup comparison (*p*  =  0.039).

## Discussion

The aim of the present study was to compare the effect of the additional use of a mouthwash containing essential oils after SRP regarding clinical outcome variables and changes of periopathogenic bacteria of the subgingival biofilm. To our best knowledge and as a result of a comprehensive search of the existing literature such a study does not exist. No unintended side effect was seen.

The 0-hypothesis can be partly rejected. The adjunctive use of the essential oil containing mouthwash gave significant better results for AL and BOP as well as for *F. nucleatum* and *T. forsythia*. The results of SRP are in coincidence with those described by Cobb et al. [3]. Cobb reported a superior result for SRP for the reduction of PD, AL and BOP as well as stronger reduction of *T. denticola*, *T. forsythia and F. nucleatum* after three or six months. Mean pocket reduction after SRP is 1.29 mm and the mean attachment level gain is 0.55 mm for pockets with PD = 4–6 mm [3]. Our results correspond to these finding. The differences of PD and AL between the groups in our study were about 0.2 mm. This seems to be low but comparing SRP with or without adjuvant antibiotic therapy or adjuvant chemotherapeutics the mean differences are between 0.06 mm for metronidazole and 0.35 mm for chlorhexidine at PD and 0.07 mm and 0.16 mm at AL, respectively, after three months [30]. Referring to findings in well-treated periodontitis patients where a further attachment loss of 0.04–0.1 mm per year occurred [31], an attachment level gain of 0.2 mm could be of importance. On the other hand it is obvious that a mouthwash with several essential oils the first days after SRP cannot have such a strong effect as a systemic antibiotic. Using a local antibiotic 17 % more pocket reduction and 12 % more attachment level gain were found in the test group in comparison to the control group with placebo [32]. The further improvements of SRP by the adjunctive use of the Parodolium® mouthwash are within the range of improvements by adjunctives to SRP [33].

It is known, that SRP reduces periodontopathogenic bacteria of the subgingival biofilm. Haffajee et al. [34] and Cugini et al. [5] reported reductions of *P. gingivalis*, *T. forsythia* and *T. denticola* while other species like *Actinomyces sp.*, *Capnocytophaga sp.*, *F. nucleatum*, *S. mitis and V. parvula* increased. A reduction of Gram negative and an increase of Gram positive bacterial species are associated with gingival health [35–38]. On the other hand, reinfections occur after several months [39, 40], already, which is in comparison to our study with exception of *T. forsythia*. The reduction of *F. nucleatum* in our study after three months is of importance because the presence of *F. nucleatum* influences further colonization of the biofilm by several periopathogenic bacteria like *P. gingivalis* and *T. forsythia* [41].

The advantage of the additional adjuvant use of the essential oil based mouthwash arises not only from the fact, that the results after SRP are only minimal smaller than those with systemic or local antibiotics, but positive antioxidative and immunoregulatory changes are described in vitro [24, 42] and in vivo [25]. Hitherto, the exact mechanisms of these effects have not be fully understood.

Over the last years, the interest in natural antimicrobials has increased and many plants have been studied for their antimicrobial properties [43]. In an in vitro study *Cymbopogon flexuosus* had the highest tested antimicrobial activity of seven in vitro tested substances with bactericidal effects against *S. aureus* and prevented biofilm formation [21]. However, no study on immunological effects of *Cymbopogon flexuosus* could be found in the literature. *Thymus zygis* has been studied more extensively from an immunological point of view. In a cellular model with human macrophages the gene expression for IL-1β, TNFα and Il-6 was significantly reduced and anti-inflammatory cytokines like IL-10 does-dependently highly increased [24]. All these cytokines are key player in periodontal inflammation. *Thymus zygis* has in vitro a proven antibacterial activity against *E. coli*, *S. enteritidis*, *S. essen* and other bacteria [42]. Within the few existing literature no results on immunological effects could be found.

Concerning the question of bias our study design reduced such a risk, as the examiner was different from the clinician performing SRP and the examiner has been anterior calibrated. The intraexaminer variability was with 0.18 mm for PD and 0.20 for AL low. The risk of bias was further reduced by using a placebo in the control group and blinding of the groups for examiner and clinician.

Further studies are recommended to generalize these findings as our study comprised only a small group of patients. However, within the limits of the study we observed positive effects especially on AL, BOP as well as on *F. nucleatum* and *T. forsythia* in the subgingival biofilm in vivo of an additional mouthwash containing essential oils in improving clinical variables as well as the composition of the subgingival biofilm in the periodontal pocket. These results indicate that in mild or moderate periodontitis cases with moderate probing depths the adjunctive use of the tested mouthwash could be useful.

## Conclusions

The adjuvant use of a mouthwash containing *Cymbopogon flexuosus*, *Thymus zygis* and *Rosmarinus officinalis* (Parodolium®) has a positive effect on the course of treatment in moderate chronic periodontitis. In particular, the significantly higher reduction of BOP and AL should be highlighted. Furthermore, the antibacterial effects should be outlined especially on *F. nucleatum* and *T. forsythia* in vivo in the context of well-known potential disadvantages of antibiotics.

## Additional file

Additional file 1: Supplementary information for the study. (PDF 36 kb)

## Abbreviations

AL: attachment level
API: interproximal plaque index
BOP: bleeding on probing
IFN-γ: interferon gamma
IL-x: Interleukin-x
PCR: polymerase chain reaction
PD: probing depth
SBI: sulcus bleeding index
SNR: signal to noise ratio
SRP: scaling and root planing
TNFα: tumor necrosis factor alpha
