# Supplementary material for: Adjunctive use of essential oils following scaling and root planing –a randomized clinical trial
Source: BMC Complement Altern Med. 2016 Jun 7;16:171. doi: 10.1186/s12906-016-1117-x (PMC4897933; doi:10.1186/s12906-016-1117-x)
Supplement: Additional file 1: — Supplementary information for the study. (PDF 36 kb) [file 12906_2016_1117_MOESM1_ESM.pdf]

Deutsches Register Klinischer Studien
German Clinical Trials Register

Trial Description

Title

Adjunctive use of essential oils following Scaling and root planing

Trial Acronym

essential oils

URL of the trial

http://nicht vorhanden

Brief Summary in Lay Language

[---]*

Brief Summary in Scientific Language

The study deals with scaling and root planing in periodontitis patients. In a randomized double-blind study we want to check if there is a benefit of the adjunctive use of essential oils during and after scaling and root planing for the improvement of clinical variables as well as at periodontopathogenic bacteria.

Organizational Data

- DRKS-ID: **DRKS00009387**
- Date of Registration in DRKS: **2015/09/30**
- Date of Registration in Partner Registry or other Primary Registry: **2012/09/24**
- Investigator Sponsored/Initiated Trial (IST/IIT): **yes**
- Ethics Approval/Approval of the Ethics Committee: **Approved**
- (leading) Ethics Committee Nr.: **332-12-24092012 , Ethikkommission an der Medizinischen Fakultät der Universität Leipzig**

Secondary IDs

Health condition or Problem studied

- ICD10: **K05.3 - Chronic periodontitis**

Interventions/Observational Groups

- Arm 1: **scaling and root planing with essential oils**
- Arm 2: **scaling and root planing without essential oils**

Characteristics

- Study Type: **Interventional**
- Study Type Non-Interventional: **[---]***
- Allocation: **Randomized controlled trial**
- Blinding: **[---]***
- Who is blinded: **investigator/therapist, assessor, data analyst**
- Control: **Active control (effective treament of control group)**
- Purpose: **Treatment**
- Assignment: **Parallel**
- Phase: **N/A**
- Off-label use (Zulassungsüberschreitende Anwendung eines Arzneimittels): **N/A**

Primary Outcome

probing depth measured with a manual periodontometer at baseline as well as after three and six months

Secondary Outcome

attachment level, bleeding on probing, periodontopathogenic bacteria

Countries of recruitment

- DE **Germany**

Locations of Recruitment

- Doctor's Practice **Hamburg**

Recruitment

- Planned/Actual: **Actual**

Planned/Actual: **Actual**

- (Anticipated or Actual) Date of First Enrollment: **2013/04/02**
- Target Sample Size: **50**
- Monocenter/Multicenter trial: **Monocenter trial**
- National/International: **National**

Inclusion Criteria

- Gender: **Both, male and female**
- Minimum Age: **40 Years**
- Maximum Age: **65 Years**

Additional Inclusion Criteria

generalized moderate periodontitis, no antibiotic use within the lst six months

Exclusion criteria

Pregnancy, breastfeeding and allergy to the ingredients of the herbal distillate products

Addresses

- **Primary Sponsor**

Institut für Mikroökologie und SymbioVaccin
35745 Herborn
Germany

Telephone: **[---]***
Fax: **[---]***
E-mail: **[---]***
URL: **[---]***

- **Contact for Scientific Queries**

MVZ Institut für Mikroökologie
Mr. Priv.-Doz. Dr. Andreas Schwiertz
Auf den Lüppen 8
35745 Herborn
Germany

Telephone: **02772981139**
Fax: **[---]***
E-mail: **andreas.schwiertz at mikrooek.de**
URL: **[---]***

- **Contact for Public Queries**

Universitätsklinikum Leipzig AöR
Mr. Prof. Dr. Dr. h.c. Holger Jentsch
Liebigstr. 12, Haus 1
04105 Leipzig
Germany

Telephone: **0341-9721208**
Fax: **[---]***
E-mail: **jenh at medizin.uni-leipzig.de**
URL: **[---]***

Sources of Monetary or Material Support

- **Institutional budget, no external funding (budget of sponsor/PI)**

Universität Leipzig
Liebigstr. 12, Haus 1
04105 Leipzig
Germany

Telephone: **[---]***
Fax: **[---]***
E-mail: **[---]***
URL: **[---]***

- **Commercial (pharmaceutical industry, medical engineering industry, etc.)**

  SymbioVaccin Gesellschaft für Diagnostik
  Auf den Lüppen 8
  35745 Herborn
  Germany

  Telephone: **[---]***
  Fax: **[---]***
  E-mail: **[---]***
  URL: **[---]***

Status

- Recruitment Status: **Recruiting complete, follow-up complete**
- Study Closing (LPLV): **2014/09/28**

Trial Publications, Results and other documents

** This entry means the parameter is not applicable or has not been set.*
